# P Wave Duration And Dispersion In Patients With Hyperthyroidism And The Short-term Effects Of Antithyroid Treatment

**Published:** 2009-09-01

**Authors:** Unal Guntekin, Yilmaz Gunes, Hakki Simsek, Mustafa Tuncer, Sevket Arslan

**Affiliations:** 1 YuzuncuYil University, Faculty of Medicine, Cardiology Department, Van, Turkey; 2 Yuzuncu Yil University, Faculty of Medicine, Internal Medicine Department, Van, Turkey

**Keywords:** Hyperthyroidism, P wave dispersion, atrial fibrillation, propylthiouracil

## Abstract

**Background:**

Prolonged P wave duration and P wave dispersion (PWD) have been associated with an increased risk for atrial fibrillation (AF). Hyperthytodism is a frequent cause of atrial fibrillation (AF).

**Methods:**

Forty-two patients with newly diagnosed overt hyperthyroidism and 20 healthy people were enrolled in the study. Transthoracic echocardiography, 12 lead surface ECG and thyroid hormone levels were studied at the time of enrollment and after achievement of euthyroid state with propylthiouracil treatment.

**Results:**

Maximum P wave duration (Pmax) (97.4±14.6 vs. 84.2±9.5 msec, p<0.001), PWD (42.9±10.7 vs. 31.0±6.2 msec, p<0.001), deceleration (DT) (190.7±22.6 vs. 177.0±10.2 msec, p=0.013) and isovolumetric relaxation times (IVRT) (90.9±11.2 vs. 79.6±10.5 msec, p<0.001) were significantly higher in hyperthyroid patients compared to control group. Pmax and PWD were significantly correlated with the presence of hyperthyroidism. Pmax (97.4±14.6 to 84.3±8.6 msec, p<0,001) Pmin (54.1±8.6 to 48.1±8.5 msec, p=0.002), PWD (42.9±10.7 to 35.9±8.1 msec, p=0.002) and DT (190.7±22.6 to 185.5±18.3, p=0.036) were significantly decreased after achievement of euthyroid state in patients with hyperthyroidism. Diastolic dyfunction was seen in 5 patients at hyperthroid state but only in one patient at euthyroid state.

**Conclusions:**

Hyperthyroidism is associated with prolonged P wave duration and dispersion. Achievement of euthyroid state with propylthiouracil treatment results in shortening of P wave variables.  Diastolic function may have a partial effect for the increased Pmax and PWD. Shortening of Pmax and PWD may be a marker for the prevention of AF with the anti-thyroid treatment.

## Introduction

Hyperthyroidism has profound effects on the heart, including increased heart rate, slightly enhanced systolic function, left ventricular (LV) hypertrophy, and diastolic dysfunction [1-9]. Atrial fibrillation (AF) is the most common cardiac dysrhythmia in the adult population. Hyperthyroidism is a major cause of paroxysmal and latent atrial fibrillation (AF) [10-12] and AF, after the sinus tachycardia, is the second common dysrhythmia in patients with hyperthyroidism. Approximately in 10 - 15 % of hyperthyroid patients AF develop [13].

P-wave duration and P-wave dispersion (PWD) are electrocardiographic (ECG) signs of prolongation of intraatrial and interatrial conduction time and inhomogeneous propagation of sinus impulses that are well known electrophysiological characteristics of the atrium prone to fibrillate [14-17].

In the present study, we aimed to evaluate P wave variables and LV diastolic function in hyperthyroid patients and to search the effects of shortterm propyl thio uracil (PTU) treatment.

## Materials and Methods

Forty two consecutive hyperthyroid  patients followed at out patient clinic of the endocrinology and metabolism department of the hospital and 20 healthy euthyroid healthy voluntary people were evaluated with 12-lead ECG and transthoracic echocardiography. Patients with systemic hypertension, diabetes mellitus, history of structural or ischemic heart disease, chronic obstructive pulmonary disease or any associated systemic disease and patients using systemic drugs, like steroids, antiarrythmics or antipsychotics, were excluded. The study was approved by the Yuzuncu Yil University, Faculty  of Medicine Ethics Committee in accordance with the Declaration of Helsinki. All participants were informed about the study and their consents were obtained.

In the patient group ECG and echocardiographic examinations were performed before treatment and after achievement  of euthyroid state with PTU treatment.

### Assessment of thyroid status

The evaluation of thyroid morphology and function was performed by clinical examination, assessment of serum free thyroid hormone T3 and T4 (FT3 and FT4) and throid stimulating hormone (TSH) levels, and ultrasound examination of the thyroid gland. A total of 5 ml of venous blood samples were drawn and then centrifuged at 2000 rpm for 10 min in a refrigerated centrifuge to separate serum. Serum FT3, FT4, TSH, levels were measured using the commercial Immulite kits, which are solid-phase, two-site chemiluminescent immunumetric assay in Immulite autoanalyser (IMMULITE, DPC, USA). The normal reference intervals were 0.80 - 1.80 ng / dl, 1.8 - 4.2 pg/ ml and 0.40 - 4.0 mIU / ml for FT4, FT3 and TSH respectively.

### Echocardiographic examination

The echocardiographic examination was performed at rest, with the patient at left lateral decubitis position, using a commercially available echocardiographic device (Vivid 3, General Electric) with a 3 MHz transducer, by two experienced echocardiographers who were blinded to the clinical data. Using M-mode echocardiography, long-axis measurements were obtained at the level distal to the mitral valve leaflets according to current recommendations of the American Society of Echocardiography [18].  The pulsed Doppler sampling volume was placed between the tips of the mitral valve leaflets to obtain maximum filling velocities. Early diastolic flow (E), atrial contraction signal (A), E/A ratio and E deceleration time (DT) were measured. Isovolumetric relaxation time (IVRT) was determined as the interval between the end of the aortic outflow and the start of the mitral inflow signal.

Tissue Doppler imaging (TDI) was acquired to assess diastolic function. The pulsed wave spectral mode was used for TDI. Filters and baselines were corrected when the velocity ranged between -20 and 20 cm/s. From the apical 4-chamber view, a 5-mm sample volume was placed at the lateral corner of the mitral annulus.  Early (Em) and late (Am) diastolic velocities were obtained. All values were measured on three separate beats and then averaged for all parameters.

Impaired left ventricular relaxation was defined as having an E/A ratio of <1 (< 0.5 for >50 years), DT > 200 msec and IVRT > 100 msec (>105 msec for >50 years), all together and/or E/Em ratio of >10 and Ea < 10 cm/sec in the presence of a preserved ejection fraction [19].

### Electrocardiographic assessment

**Figure 1:**
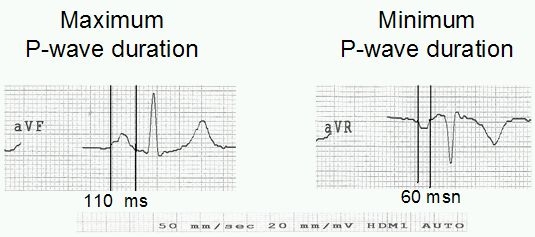
Two complexes extracted from 12-lead surface ECG of a patient with iron deficiency anemia. In this case, maximum P-wave duration was observed from lead aVF and the  minimum  P-wave duration from lead aVR. PWD was defined  as the difference between the maximum and minimum P-wave durations.

Twelve-lead ECGs were obtained at rest, with 20 mm/mV amplitude and 50 mm/sec rate with standard lead positions. ECGs were manually measured by the use of a magnifying glass by two blinded cardiologists having no information about the patients. The beginning of the P wave was defined as the point where the initial deflection of the P wave crossed the isoelectric line, and the end of the P wave was defined as the point where the final deflection of the P wave crossed the isoelectric line. The difference between maximum and minimum P wave duration was defined as PWD (Figure 1).

Intra-observer and inter-observer coefficients of variation for echocardiographic parameters and P wave variables were found to be less than 5% and nonsignificant.

### Statistical analysis

Data were presented as mean ± standard deviation (SD). Using an SPSS package 10.0 (SPSS Inc., Chicago, Illionis, USA) the changes of parameters after treatment were compared with paired t-tests. Differences in mean values between groups were assessed using the Student t test and with Mann-Whitney's U-test for variables without normal distribution. Pearson correlation analysis was used to assess the correlation between variables. A two-tailed p value < 0.05 was considered significant.

## Results

Maximum P wave duration, PWD, DT and IVRT were significantly higher in hyperthyroid patients compared to control group (Table 1). PWD was significantly correlated with presence of hyperthyroidism (r=0.501, p<0.001), IVRT (r=0.390, p=0.002), E/A (r=-0.350, p=0.005), DT (r=0.327, p=0.010), presence of diastolic dysfunction (r=0.298, 0.019) and age (r=0.255, p=0.045). Pmax was also significantly correlated with hyperthyroidism (r=0.404, p=0.001), age (r=0.270, p=0.034), E/A (r=-0.313, p=0.013), diastolic dysfunction (r=0.268, p=0.035), IVRT (r=0.265, p=0.038) and DT (r=0.259, p=0.042).

**Figure 2:**
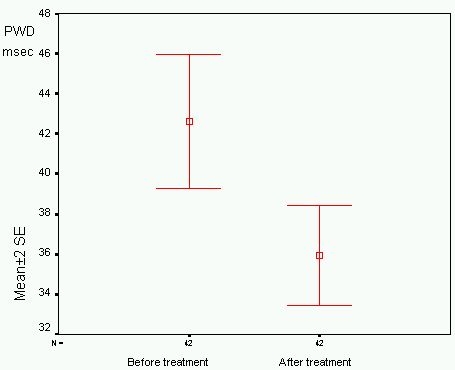
Error bar of pre- and post-treatment P wave dispersion (PWD), SE: Standard error

Euthyroid state was achieved during a 2,5±0,8 months. Pmax, Pmin, PWD and DT were significantly decreased after achievement of euthyroid state in patients with hyperthyroidism (Table 2, Figure 2). The slight decrease in IVRT was not significant. Diastolic dyfunction was observed in 5 patients at hyperthroid state but only in one patient at euthyroid state.

## Discussion

In this study we found that P maximum and PWD values were increased in hyperthyroidism and treatment with PTU results in improvement in Pmax, PWD and LV diastolic function parameters.

The correlation between the presence of intraatrial conduction abnormalities and the induction of paroxysmal AF has been well documented  [15-20]. P-wave dispersion has been advocated as a novel measurement of the heterogeneity of atrial depolarizations [21].  The increase in dispersion of atrial refractoriness may favor a re-entry mechanism leading to AF because fragmentation of the depolarizing wave front may occur when it faces adjoining atrium with nonuniform refractoriness [21,22].  Accordingly, prolonged P-wave duration and increased PWD have been reported to carry an increased risk for AF [14,15].

Frequency of AF is increased in hyperthyroidism [13]. The cardiovascular effects of hyperthyroidism result from the increased metabolic demands of the body tissues and from the direct chronotropic and inotropic effects of excess thyroid hormone on the heart. Thyroid hormone alters the action potential duration and the speed of repolarization of atrial and ventricular myocytes  [23,24]. Thyroid hormone administration shortens action potential duration and decreases the refractoriness of cardiomyocytes facilitating the maintenance of multiple reentrant circuits in hearts [25].

Increase in Pmax and PWD values have been reported in hyperthyoidism [26]. Direct effects of thyroid hormones on ionic channels and action potential [23-25] and increased sympathetic activity [27,28] may be responsible fort this finding. Recent studies established the presence of prolonged P wave duration and P dispersion as markers of increased risk of AF in hyperthyroid subjects [14,16,29]. Treatment of hyperthyroidism is frequently associated with reversion to sinus rhythm. A recent study showed that reversion to sinus rhythm occurred in 62 percent of 163 patients within 8-10 weeks after they returned to a euthyroid state [30]. Katircibasi et al  [29] demonstrated that the prolonged P wave duration and dispersion recovered with PTU treatment  at levels measured in healthy control subjects. Our results supports these findings. Therefore, anti-thyroid treatment may have preventive effects in the development of AF in patients with hyperthyroidism.

Impaired left ventricular relaxation has been reported to effect PWD [31]. It has been reported that hyperthyrodism has profound effects on cardiac diastolic function that are at least in part reversible after restoration of euthyroidism [8,32,35]. In the present study, diastolic function parameters of DT and IVRT were increased in hyperthyroid state. In addition, diastolic function was normalized in 4 of 5 patients after achievement in euthyroid state. There were significant correlations between diastolic function and Pmax and PWD. Therefore, improvement in PWD in our study may be partially explained by improvement in diastolic function.

## Limitations

Small number of study patients and short-term follow-up are the  major limitations. Another important limitation of our study is manual calculation of P-wave parameters using magnifying lens instead of computer-assisted P-wave calculations. We do not have automated measurement facilities for PWD. However, thermal, digital, and signal-averaging ECG systems have been used to evaluate PWD which was measured manually either on paper or on a high-resolution computer screen.  Although, several studies have demonstrated a low error of the measurement of P wave dispersion on paper printed ECGs [28,7]  others suggested that manual PWD measurement on paper printed ECGs obtained at a standard signal size and paper speed may have a questionable accuracy and reproducibility [36].

## Conclusions

Hyperthyroidism is associated with prolonged P wave duration and dispersion. Achievement of euthyroid state with PTU treatment results in shortening of P wave variables. Diastolic function may have a partial effect for the increased Pmax and PWD.  However, there is need for larger prospective studies to elucidate the role of P wave disturbances in prediction and early treatment of AF in hyperthyroid patients.

## Figures and Tables

**Table 1:**
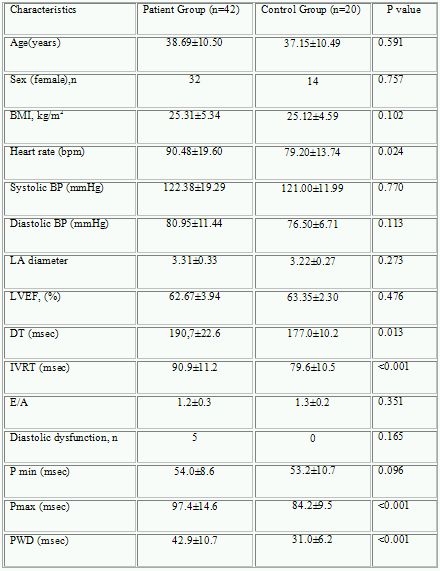
Comparison of baseline characteristics of hyperthytroid patients  with control group

**Table 2:**
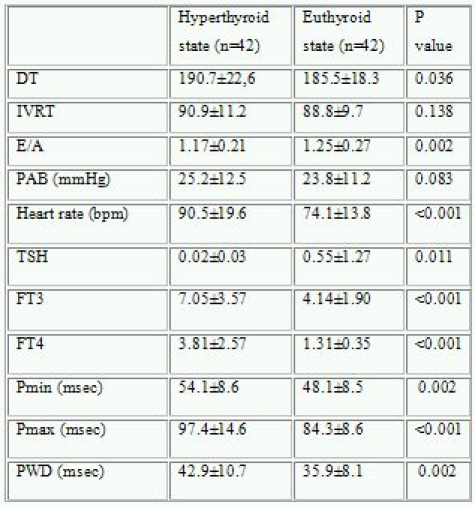
Comparison of pre- and post-treatment some echocardiographic values, thyroid hormones and P wave variables
